# An Interactive Home-Based Cognitive-Motor Step Training Program to Reduce Fall Risk in Older Adults: Qualitative Descriptive Study of Older Adults’ Experiences and Requirements

**DOI:** 10.2196/11975

**Published:** 2018-11-30

**Authors:** Trinidad Valenzuela, Husna Razee, Daniel Schoene, Stephen Ronald Lord, Kim Delbaere

**Affiliations:** 1 Physical Activity, Lifestyle, Ageing and Wellbeing Faculty Research Group Faculty of Health Sciences The University of Sydney Sydney Australia; 2 Exercise Science Laboratory School of Kinesiology, Faculty of Medicine Universidad Finis Terrae Santiago Chile; 3 School of Public Health and Community Medicine The University of New South Wales Sydney Australia; 4 Institute of Medical Physics Friedrich-Alexander University Erlangen-Nürnberg Erlangen Germany; 5 Falls, Balance and Injury Research Centre Neuroscience Research Australia The University of New South Wales Sydney Australia

**Keywords:** aged, community-dwelling, exercise, home-based training, adherence, motivation, exergame, active video games, falls, qualitative research

## Abstract

**Background:**

Falls are a major contributor to the burden of disease in older adults. Home-based exercise programs are effective in reducing the rate and risk of falls in older adults. However, adherence to home-based exercise programs is low, limiting the efficacy of interventions. The implementation of technology-based exercise programs for older adults to use at home may increase exercise adherence and, thus, the effectiveness of fall prevention interventions. More information about older adults’ experiences when using technologies at home is needed to enable the design of programs that are tailored to older adults’ needs.

**Objective:**

This study aimed to (1) explore older adults’ experiences using *SureStep*, an interactive cognitive-motor step training program to reduce fall risk unsupervised at home; (2) explore program features that older adults found encouraged program uptake and adherence; (3) identify usability issues encountered by older adults when using the program; and (4) provide guidance for the design of a future technology-based exercise program tailored to older adults to use at home as a fall prevention strategy.

**Methods:**

This study was part of a larger randomized controlled trial. The qualitative portion of the study and the focus of this paper used a qualitative descriptive design. Data collectors conducted structured, open-ended in-person interviews with study participants who were randomly allocated to use *SureStep* at home for 4 months. All interviews were audiotaped and ranged from 45 to 60 min. Thematic analysis was used to analyze collected data. This study was guided by Pender’s Health Promotion Model.

**Results:**

Overall, 24 older adults aged 70 to 97 years were interviewed. Findings suggest older adults are open to use technology-based exercise programs at home, and in the context of optimizing adherence to home-based exercise programs for the prevention of falls, findings suggest that program developers should develop exercise programs in ways that provide older adults with a fun and enjoyable experience (thus increasing intrinsic motivation to exercise), focus on improving outcomes that are significant to older adults (thus increasing self-determined extrinsic motivation), offer challenging yet attainable exercises (thus increasing perceived self-competence), provide positive feedback on performance (thus increasing self-efficacy), and are easy to use (thus reducing perceived barriers to technology use).

**Conclusions:**

This study provides important considerations when designing technology-based programs so they are tailored to the needs of older adults, increasing both usability and acceptability of programs and potentially enhancing exercise participation and long-term adherence to fall prevention interventions. Program uptake and adherence seem to be influenced by (1) older adults’ perceived benefits of undertaking the program, (2) whether the program is stimulating, and (3) the perceived barriers to exercise and technology use. Older adults shared important recommendations for future development of technologies for older adults to use at home.

## Introduction

### Background

Accidental falls are a major contributor to the burden of disease in older adults and a major public health problem. One-third of people aged 65 years and above fall every year [1]. Falls and fractures account for over half of all injury-related health care costs [2]. Personal and community burden from falls is enormous due to mobility-related disability and loss of independence. Fall-related injuries lead to a reduction in quality of life [3] and independence in self-care [4] and can precipitate admission to long-term care facilities [5]. High-quality evidence shows that well-designed exercise programs can reduce falls by 42%, provided they are continued over a period of at least 6 months [6,7].

### Adherence to Fall Prevention Exercise Programs in Older Adults

Despite the robust evidence to support exercise as an effective fall prevention strategy among community-dwelling older adults [6-9], adherence to fall prevention exercise interventions is often low [10,11], suggesting some reluctance by older adults to take part in such programs [11,12]. Systematic review evidence indicates that adherence rates to home-based exercise programs for fall prevention are initially high at 82% over the first 2 to 4 months, but rates drop down to approximately 52% over 1 year [11].

A review of older adults’ perspectives of fall prevention exercise programs found that social interaction was frequently suggested as a motivator to participate in group-based programs [13]. Qualitative data also suggest that exercising in the company of others provides older adults with pleasure and motivation to continue, and for some, social interaction is the primary reason for participating in such programs [14]. However, although some older adults value the professional supervision and social interaction and encouragement by peers [15,16], others perceive many barriers to participation in group programs. The existing evidence suggests that not all older adults wish to participate in group-based programs and that some older adults perceive it as a barrier to exercise participation. Examples of common barriers to attend group exercise programs away from home include the variety of skill levels within the group [14]; the requirement of assistance for some participants, meaning that others have to wait [14]; feeling uncomfortable about having the lowest skill level or not feeling equal within the group [14]; feeling intimidated to attend fitness facilities and other group exercise settings [17]; and environmental barriers such as lack of transport [18,19], effort and costs associated with traveling [20,21], adverse weather conditions [15,22], and the need to care for an ill spouse [19]. Furthermore, factors including age older than 80 years, poor self-reported health status, recurrent falls, concerns about falls, and avoidance of activities have also been associated with an increased preference to undertake home-based programs [23]. Moreover, some older adults simply prefer the convenience, privacy, and autonomy that home-based exercise programs provide [21,24]. Technology has been used to address these barriers and promote exercise participation. This study is important in providing in-depth qualitative insight into older adults’ experiences when using a technology-based fall prevention exercise program in their homes. Recommendations made by older adults will be valuable in shaping the development of future technologies to provide fall prevention interventions in older adults’ homes.

### Use of New Technologies to Deliver Home-Based Exercise Programs for Older Adults

Technology-based exercise programs (also known as “exergames”) offer several advantages over traditional exercise programs in that they can contribute to a more enjoyable and stimulating exercise experience. Some advantages include the opportunity to tailor the exercise modalities according to the abilities of each individual (balance, strength, functional exercises, etc), provide individualized progression of exercises by unlocking levels of difficulty according to each individual’s performance, offer a wide variety of exercises to maintain engagement, provide users with reinforcement and real-time feedback while exercising, and monitor performance over time [25-27]. Exergames also allow the introduction of a fun gaming factor to enhance motivation and increase participation [28] and can offer combined training of sensory, cognitive, and physical functions by changing the tasks displayed on the screen [29]. These factors are important as declines in attention, psychomotor processing, and problem-solving have an important impact on postural control, gait, and falls in older adults [30,31].

Several systematic reviews have shown that it is safe and feasible for community-dwelling older adults to engage in exergames [32-37]. Furthermore, it has been reported that older adults report higher levels of motivation and engagement when exercising using exergames [33] and using exergames may also enhance social well-being [38]. Technology-based exercise programs have shown comparable improvements in physical function when compared with other exercise programs [36,39]; systematic review evidence suggests that older adults’ adherence to technology-based exercise programs is similar—or slightly better—to traditional exercise programs [40].

These results suggest that technology-based exercise programs may be an effective alternative or complement to conventional exercise programs. The possibility of using these programs in older adults’ homes may further increase exercise participation in the proportion of the community that is either unable or unwilling to take part in other exercise programs. However, it is important to consider that most randomized control studies in this area have been conducted under close supervision in laboratory or clinical settings, using mostly commercial game consoles such as Nintendo Wii console, Xbox, and PlayStation [40] that are not specifically designed for older adults. Factors such as the pace of gameplay, the amount of graphical information, and the instructions on how to use the program can make it difficult for older adults to use these programs on their own [25,27,36,37].

Only few randomized controlled trials (RCTs) have tried to overcome these usability problems and improve exercise adherence to home-based interventions by using technology-based exercise programs that have been custom-developed for older adults [25,41-43]. Results from these studies are encouraging as older adults have been able to independently use these technologies in their homes, and interventions have been effective at improving physical [41,42] and cognitive parameters of fall risk in older adults [41,43]. The implementation of technology-based exercise programs for older adults may increase exercise participation and adherence and, therefore, has the potential to increase the effectiveness of fall prevention exercise programs. However, as the implementation of custom-developed technologies for older adults is in its early stages, more information about older adults’ experiences when using technologies independently in their homes is needed.

### Description of Intervention: SureStep—a Home-Based Interactive Cognitive-Motor Training Program for Fall Prevention in Older Adults

This study is part of a larger RCT [41,43]. The purpose of the RCT was to assess the feasibility and safety of older adults using *SureStep* —an interactive step training system designed for older adults to use unsupervised in the home setting—and determine the effectiveness of this intervention on stepping performance and associated fall risk in older adults compared with a nonexercise control group. The study showed that 16 weeks of unsupervised interactive cognitive-motor training using the *SureStep* system led to improvements in specific cognitive functions associated with falls in older people [43].

**Figure 1:**
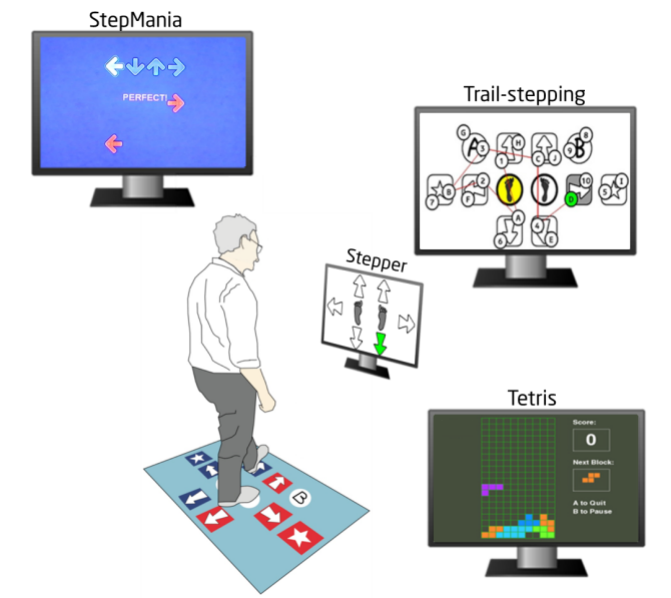
Schematic representation of SureStep: an interactive cognitive-motor step training program for fall prevention in older adults.

*SureStep* consists of a step pad that is connected to a computer unit and a television to present cognitive-motor training stimuli. The motor (stepping) component of *SureStep* aims to train people to take quick and accurate lateral and anterior-posterior steps, and the cognitive component, delivered as engaging *games*, aims to train specific executive functions including working memory, visuospatial skills, dual-tasking, inhibition, and attention. The combination of step training and video games makes it possible to increase the training complexity by adding challenging cognitive tasks. *SureStep* consists of 4 games (*StepMania*, *Stepper*, *Trail-stepping,* and *Tetris*) that are modified versions of popular video games (Figure 1). Although the nature and objectives of the step exercises (games) differ, the basic action of making well-timed and directed steps to solve tasks is common to all games. Games were designed to provide the user with feedback on their performance and multiple levels were available, with the harder levels requiring higher cognitive capacity and physical effort to perform the tasks.

Participants were instructed on the use of the system during individualized sessions at the start of the study. In addition, participants received a manual with detailed instructions regarding the use of the system. To facilitate compliance and to resolve any difficulties with system use, participants were telephoned at the end of weeks 1, 4, 8, and 12. Participants could also call the research team at other times if required and additional home visits were offered if requested. Participants were asked to play each game at least once during each session as many times as they wished, with the recommended dose of 3 sessions of 20 min per week during the 16-week trial. The time and duration for system use and game performance were recorded, saved by the game computer, and uploaded to a custom-made website by the system. Participants not using the system for 2 consecutive weeks were contacted by telephone to solve any issues and encourage adherence. Adherence was measured using the recorded logs of the system use.

Participants required on average 2 instructional visits of 90 min each (mean 2.0, SD 1.2). During the 16 weeks of intervention, participants played on average 31.8 sessions (SD 21.9) with a mean duration of 27.4 min (SD 28.1) for a total of 1317 min (SD 2075). A total of 18 participants achieved the target of 960 min (16 weeks, 3 sessions per week, 20 min); however, only 1 participant performed each of the 4 tasks at least 3 times per week over 16 weeks. During the trial period, 32% (15/44) of the intervention participants withdrew or stopped training. Technical problems with the step training system led to 3 participants ceasing training and interfered with the training dose of others. Other reasons for withdrawal included death (n=1), medical reasons (n=8), and personal reasons (n=3).

A comprehensive description of the interactive cognitive-motor step training program *(SureStep)* as well as the RCT and its effectiveness can be found in the protocol and main outcome papers [43,44]. An instructional video of the *SureStep* cognitive-motor training system can be found in Multimedia Appendix 1.

### Research Aims

This paper reports on the qualitative findings from participants who were randomly assigned to use *SureStep* for 4 months as part of the larger RCT. The aims for the qualitative portion of the study were as follows: (1) to explore older adults’ experiences using *SureStep*, an interactive cognitive-motor step training program to reduce fall risk unsupervised at home; (2) to explore which program features older adults considered encouraged program uptake and adherence; (3) to identify usability issues encountered by older adults when using the technology independently at home; and (4) to provide guidance for the design of a future technology-based exercise program tailored to older adults to use independently at home as a fall prevention strategy.

## Methods

### Study Design

A qualitative descriptive design was used [45,46]. Qualitative description is based on the theoretical foundation of naturalistic inquiry, which aims to study events and persons in their natural state. The methodology aims to provide an accurate description of the phenomenon using everyday language. Pender’s Health Promotion Model (HPM) [47] was used as a theoretical framework to help design the research inquiry and guide the development of the interview. This study was approved by the Human Research Ethics Committee of the University of New South Wales (Reference number HREC 12316).

### Sample

Participants were selected from the intervention group participants that completed the *SureStep* training program. To ensure a range of viewpoints, maximum variation sampling [48] was used to purposively select participants that reflected different ages, gender, health status, familiarity with technologies, and adherence to the exercise program (N=24). Residents of independent-living units of a retirement village in Sydney and from the community who met the following criteria were eligible to participate in the larger RCT: (1) aged 70 years or above; (2) living independently; (3) able to walk with or without a walking aid; (4) able to step unassisted on a step pad; and (5) have no severe lower extremity pain. Exclusion criteria were major cognitive impairment (Mini-Cog<3), diagnosis of degenerative disease, color-blindness, corrected vision of at least 6/16, or an unstable health condition.

### Data Collection

Structured interviews with open-ended questions were conducted within 30 days of participants completing the 4-month intervention using *SureStep*. The interview guide was pilot-tested with 3 older adults before administration to refine wording of the questions; these were not used in the analysis. The interviews focused on understanding older adults’ motivations to use the *SureStep* program, perceived benefits of using the program, barriers and enablers to exercise participation and adherence, as well as program usability. The interview guide used at completion of the 4-month intervention period can be found in Multimedia Appendix 2.

Interviews were conducted by the same researcher who instructed participants on the use of *SureStep* and provided assistance throughout the 4-month intervention study. This likely encouraged participants to talk more openly about both the positive and negative aspects of their experience (eg, technical difficulties they experienced) with someone familiar to them and who was aware of the technical difficulties some participants had experienced. During the training program, monthly logs of telephone contact and home visits, as well as detailed field notes from the home visits detailing problems encountered by each participant were maintained. These were used for data triangulation [49] as well as in the analysis to address the technical difficulties participants experienced when using the program unsupervised in their homes.

Following informed consent, interviews were audiorecorded to allow verbatim transcription and subsequent analysis of the data. Member checking [49] was performed at the end of each interview to ensure accuracy in data collection by summarizing the initial findings to the participant and gaining confirmation that the summary reflected their views, feelings, and experiences. Participants were also given the opportunity to add further information. Interview length varied between 45 and 60 min each. Collection of data continued until data saturation was achieved and no new information was revealed [49]. Interviews were transcribed verbatim by a third person not involved in the study and verified. All participants chose to have the interviews conducted in their homes.

### Data Analysis

Interview transcripts were analyzed using thematic analysis [48,50]. The data were analyzed by TV and HR. Initially, transcripts were read to become familiar with the data. Then, the transcripts were read again highlighting text that appeared to describe an emotional reaction to using the *SureStep* program. This was documented with a keyword that captured the emotional reaction—using the participant’s own words—on the margin of the text. After open-coding 4 transcripts, authors met to cross-check information and discuss any discrepancies. A consensus list of preliminary codes and a specified definition for each code was then generated and used as a guide for further coding. Transcripts were then imported into a computer software program (NVivo version 10, QRS International Pty, Doncaster, Victoria, Australia) to help manage the data and maintain an audit trail [51] of the steps and decisions taken during the analysis process. Throughout the coding of the interview transcripts, regular meetings took place to ensure rigor through a process of investigator triangulation [49]. As the coding progressed, some codes were combined, whereas others were split into subcategories. Final codes were then examined and organized into a hierarchical structure when possible. During data coding, the technique of constant comparison [48] was used to compare and contrast the categories within and across participants of different age groups, experience with the use of technology, level of physical activity, and self-reported health status. This enabled exploration of the relationship between participants’ characteristics and their experience using the program.

## Results

### Older Adults’ Demographic Characteristics

All invited participants agreed to take part in the study. Overall, 24 interviews were conducted. Participants’ demographic and health characteristics are presented in Table 1. Out of the 24 interviewees, 74% (17/24) were women and ages ranged from 70 to 97 years. All participants resided in the Sydney metropolitan area; 78% (18/24) of the participants lived in independent apartments within a retirement village and 48% (11/24) of participants lived alone. Participants had a low number of comorbidities, were cognitively intact, and had low levels of depressive symptoms. Over half of the participants (65%, 15/24) had experienced a fall in the previous year and 30% (7/24) used walking aids when outdoors. Approximately half of the participants (48%, 11/24) reported having some previous experience using computers, and only 1 participant had previous experience using a commercial exergame (Nintendo Wii). Baseline self-reported levels of physical activity showed that 50% (12/24) of the participants were adhering to the general World Health Organization recommendation of 150 min of exercise per week [52]. Of the 24 participants interviewed, 18 participants (75%, 18/24) met the target recommendation of 960 min of exercise throughout the 4-month intervention period (20 min, 3 days per week, 16 weeks). Total time played over the 16-week intervention period ranged from 3.3 hours to 205.2 hours (mean 35, SD 47.6 hours). Reasons for not meeting the recommended exercise dose included prolonged hospitalization (n=2), pain that was aggravated by exercise (n=1), disinterest or lack of time (n=1), and difficulty using the system (n=2). Further follow-up visits were provided to the 2 participants who did not meet exercise recommendations due to technical difficulties, and further step-by-step instructions on program use were also provided. Participants were able to use the system under the supervision of the research staff; however, their inability to remember the instructions when unsupervised led them to stop using the program before the end of the intervention period.

### Qualitative Findings

Analysis of the interview transcripts generated 3 main themes that reflect older adults’ motivations as well as experiences and perceptions of using the *SureStep* program, as illustrated in Textbox 1. The results are presented according to the following 3 themes: (1) “It must be beneficial,” (2) “It must be stimulating,” and (3) “It must be accessible.” The words in italics or in quotes are sentences or expressions as spoken by the participants. Pseudonyms are used when making a reference to study participants to ensure participants’ confidentiality.

**Table 1:** Characteristics of the participants at study baseline.

Characteristics		Subsample of interviewed participants (n=24)^a^	Total sample of intervention group participants in the RCT^b^ (n=47)
Age (years), mean (SD)		81.4 (7.5)	82 (7)
Female, n (%)		17 (74)	31 (66)
Body mass index in kg/m², mean (SD)		26.3 (5.5)	27.1 (5.9)
Level of disability (WHODAS^c^ 0-48), mean (SD)		15.6 (3.5)	17.1 (5.1)
Comorbidity (FCI^d^ 0-18), mean (SD)		3 (2.2)	3.55 (2.2)
Number of medications, mean (SD)		4 (3.4)	4.55 (3.3)
Overall cognition (Mini-Cog), mean (SD)		4.3 (0.8)	4.4 (0.8)
Depression (PHQ^e^-9), mean (SD)		2 (2.5)	2.9 (4.1)
Concern about falling (Icon-FES^f^), mean (SD)		48.3 (14.2)	53.9 (18.2)
Falls in the past year, n (%)		15 (65)	18 (38)
Use of walking aid outdoors, n (%)		7 (30)	13 (28)
Use walking aid indoor, n (%)		1 (4)	4 (9)
Computer literate, n (%)		11 (48)	—^g^
Resident of retirement village, n (%)		18 (78)	37 (78)
Single person in the household, n (%)		11 (48)	—
**Total physical activity, hours per week (IPEQ^h^), mean (SD)**		25.3 (15.1)	26.4 (14.6)
	Planned Physical activity (hours/week), mean (SD)	2.8 (2.4)	2.6 (3.6)
	Perform ≥150 min per week, n (%)	12 (52)	49 (54)
	Incidental physical activity (hours/week), mean (SD)	21.9 (14.8)	22.1 (14.2)
Participants meeting recommended total exercise intervention dose of (960 min over 16 weeks), n (%)		18 (75)	18 (38)

^a^Values are for 24 participants except for body mass index, weight, and height (N=20) and IPEQ (N=22).

^b^RCT: randomized controlled trial.

^c^WHODAS: World Health Organization Disability Assessment Schedule.

^d^FCI: Functional Comorbidity Index.

^e^PHQ-9: Nine-item Patient Health Questionnaire.

^f^Icon-FES: Iconographical Fall-Efficacy Scale.

^g^Dashes indicate that data was not collected.

^h^IPEQ: Incidental and planned exercise questionnaire for older adults.

**Themes and subthemes generated from the interview transcripts.**

It must be beneficial

- Improving physical and cognitive functioning
- Being of help to others

It must be stimulating

- Feeling physically and mentally challenged
- Importance of exercise variety and difficulty levels
- Seeing progress being made

It must be accessible

- Benefits of home-based delivery
- Program design and usability

#### Theme 1: It Must Be Beneficial

This theme relates to the different self-determined extrinsic motives by which older adults engaged in the *SureStep* training program. The interviews revealed that the main reasons why participants adhered to the program were to improve their physical and cognitive function and to help others.

##### Subtheme 1.1: Improving Physical and Cognitive Functioning

A feature that distinguishes *SureStep* from more conventional forms of exercise is that *SureStep* exercises were designed to provide older adults with combined cognitive and motor training. It was clear that participants appreciated this feature as they said *SureStep* was training both “their body and their mind.” When participants were asked about their motivations to use *SureStep*, it was apparent that those who had lower self-reported health status, poorer balance, and higher fear of falling wanted to do it “for [themselves]” as they thought that exercising would be “beneficial for them.” They seemed to be more conscious about the detrimental effect that inactivity can have on older adults. For example, they spoke of being aware of the “debilitating effects of lack of mobility,” which some described as “getting slow at doing things,” “losing [their] balance,” and “falling.” For Robert (aged 81 years), typical of the majority of study participants, having a fall represented a threat to his independence; thus, “preventing falls” and “remaining independent” were 2 of the most important motivators to take up and adhere to *SureStep*.

Perceived benefits attributed to using *SureStep* were the belief that using the program would help to *keep [them] healthy* and *improve [their] abilities,* including their *balance*, *reaction time, ability to walk further,* and *stay fit.* The perceived value of using *SureStep* seemed to rely on the belief that improving, or at least maintaining, their physical abilities would allow them to continue living independently for longer as portrayed in Thelma’s words. Thelma’s words also clearly indicate how important living independently is for these participants:

[...] if you can save yourself from falling or tripping, you can still have your own shower; you can still get your own meals; you can still take yourself out and about. It’s worth everything to the individual to be able to do that. It doesn’t matter how old you are, being able to do that is just marvellous.

Thelma, 87-year-old female

For this group of participants, adhering to the *SureStep* training program was helping prevent cognitive decline and to keep their mind and body active:

Well, I knew it would help me think faster and move faster. You don’t want to lose your faculties, you want to be able to remember people’s names, you want to be able to remember where you’re going and what you’re doing and remember things that happen.

Agnes, 85-year-old female

Some older adults seemed to feel an aversion to aging, as they feared they would lose their physical and cognitive capacities. The desire to *delay* the effects of aging came through in Bernice’s words:

I thought it will keep me younger if I keep using my mind and my legs and arms [by doing SureStep training program].

Bernice, 89-year-old female

Higher adherence rates were attained by participants with lower self-perceived health status, poor balance ability, and fear of falling and by participants who were hoping to obtain health-related benefits. The improved physical and cognitive functioning participants spoke of reflects the notion of perceived benefits, which, according to HPM [53], helps promote positive health behavior. Even though on commencement of the study, adherence rates were mostly high for all participants, adherence rates for some participants declined over the duration of the trial. When participants were asked why their adherence declined, it was apparent that participants’ motivation to continue their engagement over time relied on them perceiving that they *needed* to perform the exercises. The notion that people adhere to actions from which they derive personally valued outcomes, such as health benefits, comes through in Donald’s words:

[Interviewer: you said towards the end of the program you lost interest in the exercises, what do you think could have helped you stay motivated?] Yeah. Yes. The only thing I can think of is if—I was finding I was losing my physical fitness—that would motivate me because I’m going from here to the gym a few days still...I think that—yes, once people see they’re getting something out of it, yes, then you would take it on board. Yep. And that’s one of my problems, I can stand on one leg.

Donald, 78-year-old male

##### Subtheme 1.2: Being of Help to Others

In addition, 3 participants, all of whom rated their health and balance ability as good or very good, reported that even though they hoped to improve their physical and cognitive abilities using *SureStep*, their main motivation to engage in *SureStep* was not for health-related motives, but rather for the value they put on helping other people by contributing to research. Participants felt that by becoming research participants, they were helping researchers get the numbers they needed and thereby, in future, helping others:

It was possibly helping somebody else that might be in an area where they are going to have problems with mobility. I think that was—that would be the number one driver as to why I did it; that it might benefit somebody that has had falls or suffered from some problem.

Ron, 81-year-old male

Ron who during the interview indicated he did not see himself as someone who was yet at risk of falling, was adhering to the program to help others as clearly reflected in his words. Similar to Ron, 78-year-old Donald who had described himself as being quite healthy and active was “committed to the program [SureStep]” to help with the research, “mainly because you need the numbers and you’re not getting them.”

It appears, overall adherence rates during the 16-week intervention period were higher in the group of participants whose main reasons to use *SureStep* were to improve their physical and cognitive function (n=17, mean time played 2103, SD 2859 min) when compared with those whose main reason was to help others by contributing to research (n=3, mean playtime 1073, SD 158 min).

#### Theme 2: It Must Be Stimulating

This theme relates to various features of *SureStep* that increased older adults’ intrinsic motivation to engage in the training program. The interviews revealed that participants found the exercise experience enjoyable and stimulating when they felt physically and mentally challenged, when there was variety in both exercises and difficulty levels, and when they could see that they were making progress.

##### Subtheme 2.1: Feeling Physically and Mentally Challenged

Participants described *SureStep* as being *fun* and *challenging.* Ada’s words below suggest that what distinguished *SureStep* from other forms of exercise was the design of the program, which challenged both cognitive and motor tasks simultaneously preventing users from getting bored:

Well going to the gym bores me to tears, walking bores me to tears, that [SureStep program] keeps me active, my mind as well as my body and that is the main thing I enjoy.

Ada, 76-year-old female

For participants of this study, such as Doris (93 years), there were not many things that would present a challenge to them in their current daily activities. Engaging in *SureStep* was appealing as it presented a challenge to their otherwise routine life:

Well, I think it’s a challenge mentally for you to do it because as you get older there’s not that many challenges that make you think—that makes you think quicker...Yep. Because it’s very easy to let things slide, you know. You don’t challenge yourself very much, particularly when you’re living by yourself. If you have another person living with you they’re challenging you but if you’re living by yourself there’s no challenge. So that was a bit of a challenge [referring to the exercise program].

Doris, 93-year-old female

##### Subtheme 2.2: Importance of Exercise Variety and Difficulty Levels

Participants expressed that having a *variety* of exercises and *different difficulty levels* was a good way to keep them engaged with the program as it made the experience more *interesting* and also made them want to *keep going from easy to more difficult and more difficult*. Furthermore, the ability to progress through different levels of difficulty made them feel *proud* and *good* about themselves and at the same time provided a *fun* way to exercise. These findings also suggest that older adults’ self-efficacy and perceived competence to exercise with *SureStep* increased as they progressed from easier levels of gameplay to harder levels as depicted in Anna’s words:

Well, because if you are good at one level, then you can go up to the next. And then you feel, oh look you’ve done so well. Then you go up to the next one. And then you feel good. And you walk around saying, I did this and I did that.

Anna, 72-year-old female

The importance of varying difficulty levels was expressed by participants of different ages, perceived health status, and balance ability.

However, despite most participants finding the program fun and challenging, 3 participants found that as the weeks passed, the *novelty* wore off and they started to get bored with the exercises, which reduced their motivation to use the program. All 3 participants rated their balance to be “good” or “very good,” had low concern about falling, were engaged in other activities (such as social activities, minding grandchildren, and doing voluntary work), and took part in other forms of exercise, for example, walking, going to exercise classes, and attending the gym. Two examples are the experiences of Ron and Donald:

After you have done it for a couple of months, you sort of think, well, I have done that one, I have done another one [referring to the games]—and I can go up to level hard and that is about as far as I can go.

Ron, 71-year-old male

Like Ron, as time passed, 78-year-old Donald found he was “running out of challenge” and he “wasn’t going to progress any further, [he] had reached the top,” making Donald lose interest in the program.

For participants like Ron and Donald, their intrinsic motivation to use the program (ie, because it provided a fun and challenging experience) decreased as time passed, and instead, their motivation became driven by nonself-determined sources of motivation: they felt they *had* to do it because they had *committed to participating in the research.*

The loss of interest in the program due to lack of novelty did not prevent these participants from reaching the recommended exercise dose. However, it is important to consider that they all reported continuing to use *SureStep* because of their commitment to the research study. Thus, under other circumstances, a lack of novelty could lead to reduced adherence rates and program discontinuation.

Participants suggested that to prevent people from getting bored with the exercises and motivate them to continue, new games could progressively be introduced to the program to maintain *novelty*. The need to have a larger variety of games to maintain the challenging nature of the exercises and with that the feeling of enjoyment in the activities reflect the “activity related affect” construct of HPM. That is, older adults are more likely to engage and continue a behavior, such as exercise participation, if they associate the behavior (performing exercise) with positive feelings and emotions. However, when participants got used to the exercises, the novelty and challenge wore off and with that enjoyment.

##### Subtheme 2.3: Seeing Progress Being Made

Designing exercise programs in a way that older adults can see that *they are improving, they have made progress* was seen by all participants as an essential feature of the *SureStep* program. Before starting the program, some participants, particularly those with low self-reported health status and balance ability and moderate to high levels of fear of falling, questioned whether there was any point in starting an exercise program. They said “what’s the good of me doing it [referring to exercise in general],” as they thought they were already *too old*, *too slow,* and *not [sufficiently] well* to exercise. However, after having exercised with *SureStep* and having seen that they could progress to harder levels, they felt *good* and proud about themselves (eg, *I did this and I did that)*, improving their self-esteem*.*

Participants indicated that feedback on their performance such as getting a score for their performance was *a great way of encouraging people to continue to exercise*. For 78-year-old Eva, getting a score motivated her to compete against herself *to get a bigger score* and this *made exercising fun*.

When comparing playtime between the 4 available games, it was apparent that *Stepper* was the least preferred game. Participants attributed this to the lack of feedback from *Stepper* game:

[…] it doesn’t tell you anything, you don’t know if you are doing better or not. The others give you a score or tell you how long you are takin to solve it you know? There is no challenge in this one.

Ada, 76-year-old female

Feedback is, therefore, an important component of technology-based exercise programs, as it makes older adults feel they can still achieve their goals and *help themselves.* This is reflective of the construct of self-efficacy in HPM. According to this theory, the higher older adults perceived competence or self-efficacy to perform the *SureStep* exercises, the higher is the likelihood that they would commit to participating. Self-efficacy, in turn, was increased when participants saw they could successfully perform the exercises and improve their scores. According to HPM, greater self-efficacy results in fewer perceived barriers to exercises. Thus, the less likely it will be that feelings of *being too old*, *too slow,* and *not well* will interfere with their commitment to engage in exercise.

#### Theme 3: It Must Be Accessible

This theme relates to the preference of participants to have a home-based exercise program and the importance of designing technology-based exercise programs that older adults can easily use independently in their homes. The interview responses revealed that participants liked having a home-based exercise program as it reduced common barriers to exercise. However, difficulties using *SureStep* led to 2 participants stopping the use of the program and other feeling frustrated with the system. This suggests that further development is needed to make the program more user-friendly for older adults to use unsupervised.

##### Subtheme 3.1: Benefits of Home-Based Delivery

When participants were asked about their perception of having *SureStep* as a home-based program, most participants conveyed that being able to exercise in their homes was very “convenient,” as it allowed them to exercise “when they liked and for as long as they liked” without having to be constrained to a schedule. Such convenience was important as many of these participants had a variety of caring, volunteering, social, and medical routines that impacted their availability to attend center-based exercise programs. For Gertrude (aged 97 years), a home-based program gave her flexibility as she poignantly expressed:

This had all the elements that suited me. Because it was in my own home, because I didn’t have to answer at half past six in the morning, because I could see and adjust myself to what was presented on the TV screen [referring to exercises], all that was going my way. I had often looked at—because there’s four or five courses going all the time in this place, I’d often looked at them and thought no, I cannot make this body get up and be sure I’ll be there half past eight every morning, which is what some of them require. So you see if it rains I can't go anywhere, because I can't take the scooter out in the rain and I can’t walk. And buses only come at certain times, not when it suits you. So that I - this was absolutely handmade for me.

Gertrude, 97-year-old female

The importance of having the *SureStep* program as an “all-weather capability of moving your body around” comes through clearly in Gertrude’s words.

Being able to exercise in the *privacy* of their home was especially important to those who felt self-conscious about their bodies and their reduced abilities, as participants considered exercising in their home to be more *personal.*

Although most participants preferred to exercise in their home, for 3 participants, this was seen as a barrier to exercise participation, as they preferred other modes of exercise. For George (aged 87 years), who enjoyed being outdoors, using the program in his home was not appealing:

Well, standing in front of the television and going backwards and forward, I get bored. Because before that and now, I generally go [out] every day, I go for a walk, I go through the bush. So I’d prefer going out. Because I like to get outside, so I’d rather go outside and do things outside and walk around.

George, 87-year-old male

Marion (86-year-old female), on the other hand, thought that joining an exercise class within the village would help her adhere to the program, as committing to a class would mean she had to attend, whereas at home she could “cheat and gradually stop exercising.”

For 84-year-old Evelyn, the social aspect of exercising seemed to be very important. She preferred to make exercise *a social thing* and thought it would be a good idea to set up the stepping mat exercise program in the retirement village, so people could go and exercise together.

In the context of exercise adherence, the higher the number of perceived barriers to exercise, the lower the likelihood is that individuals will adhere to an exercise program. This is in accordance with the perceived barriers to action construct of HPM.

##### Subtheme 3.2: Program Design and Usability

When designing technology-based exercise programs for older adults, it is important to take into account specific usability aspects of program design, as these can become a barrier to participation, especially for those without previous experience using technology. This subtheme is divided into participants’ experiences navigating through the program and the technical difficulties experienced.

#### Participants’ Experiences Navigating Through the Program

Participant accounts of their experiences when interacting with the *SureStep* program were varied, with some older adults finding it easy to use and others very difficult. When contrasting participants’ experiences, it became apparent that those who described the program as being *quite easy to use* and *self-explanatory* were all accustomed to using other technologies (eg, computers, DVD player, and tablet computers), and they themselves talked about how their previous experience with technology may have facilitated the use of *SureStep*:

It was only a few days [before I became familiar with the program]—see, I’d worked on computerised stuff for 20 odd years on different programs, different workings, different—seen how programs worked and that [SureStep] was probably much easier for me than [for] some other people.

Rubi, 78-year-old female

Although participants like Rubi found navigating through the program very easy, others experienced difficulties when trying to select a game, change from one game to another, and move between difficulty levels, which led to feelings of frustration. This is portrayed by Betty (aged 80 years), who described herself as “not being computer savvy,” but rather a “luddite” when it comes to using technology:

Well I know it drove me mad because, I obviously, I grew to understand it [referring to how to navigate through the program]. But at the beginning when I didn't know that if I did that, that would fix that, so I had a lot of frustration at the beginning. Well I think it was mostly my fault, the problem was that I wasn’t understanding computers. It might have taken me the first month [to learn how to navigate through the programs] because I know I fought with it for a long time.

Betty, 80-year-old female

Some participants, particularly those aged 80 years and over and those with low technology familiarity, spoke of having trouble using *SureStep* as they would often forget how to use the program. Participants were given a booklet with detailed instructions on how to operate the program; however, some did not use it because they “had forgotten [they] had it” or thought it was a “nuisance” having to stop and get the book because that “used up the time [they] had to exercise.” The frustration participants felt when forgetting how to operate the program is portrayed in Gertrude’s words:

It irritated me that I, 'cause I get irritated with myself, that I couldn’t go straight into and do it, because I think my short term memory is a little bit weak and therefore, I was forgetting what you told me, by the time I tried to put it into practice. But that will happen to anybody of my age.

Gertrude, 97-year-old female

Gertrude’s experience was quite typical of the difficulties that the study participants experienced when navigating through the program to select an exercise game, change from one game to another, and move between difficulty levels. These were all common sources of frustration for some participants and can act as perceived barriers to action.

#### Technical Difficulties

Participants encountered similar problems when navigating through the program interface. Again, this was more prevalent among participants aged 80 years and over and those who had limited experience using technologies. Some participants felt frustrated when unable to find a particular game they wished to play on the home screen. From observations made during home visits, it became evident that some participants were unable to remember that there were other game options in addition to what was presented on the screen and that by stepping right or left on the step mat they could access them. Future designs should ensure that all games are displayed on the home screen.

Similarly, participants reported often forgetting how to select a game from the home screen, how to return to the screen to change the level of difficulty of the game, and how to exit the game to play another game. The instructions were not consistent between games and participants said they would like to have all 4 games “work the same way” and have “explicit step by step instructions” on the screen to guide users in the operating of the program. This comes clearly in Eva’s words:

I think perhaps the instructions could have been a bit more explicit, yes...I think it could have been a bit more, a bit more basic, not presuming that everybody could use computers...For non-computer literate people- and you’re going to get a lot of them in this age group. A step-by-step [instruction] could have been better.

Eva, 78-year-old female

Finally, some participants also experienced technical problems such as the program temporarily not responding to the step mat or the program not starting correctly when turning it on. This impacted participants’ willingness to use the program as portrayed by Thelma:

I think that sort of put you off a bit [the program not functioning properly] and I thought oh well I won’t do it now, it won't work. I haven’t got time now to fiddle with it [...] you almost lost interest in it [the program] because it didn’t operate immediately. It didn’t matter what you did. And you thought, oh I haven’t got time now and that’s what you do.

Thelma, 87-year-old female

Difficulties were also encountered when moving from one game to another or changing difficulty levels, as the program would sometime flick through the different options too quickly. This was only experienced by a couple of participants due to faulty equipment and resolved after replacing the mat. However, this shows that it is of crucial importance to optimize the design and development before providing it to older adults, especially to those with limited prior experiences with technology.

**Figure 2:**
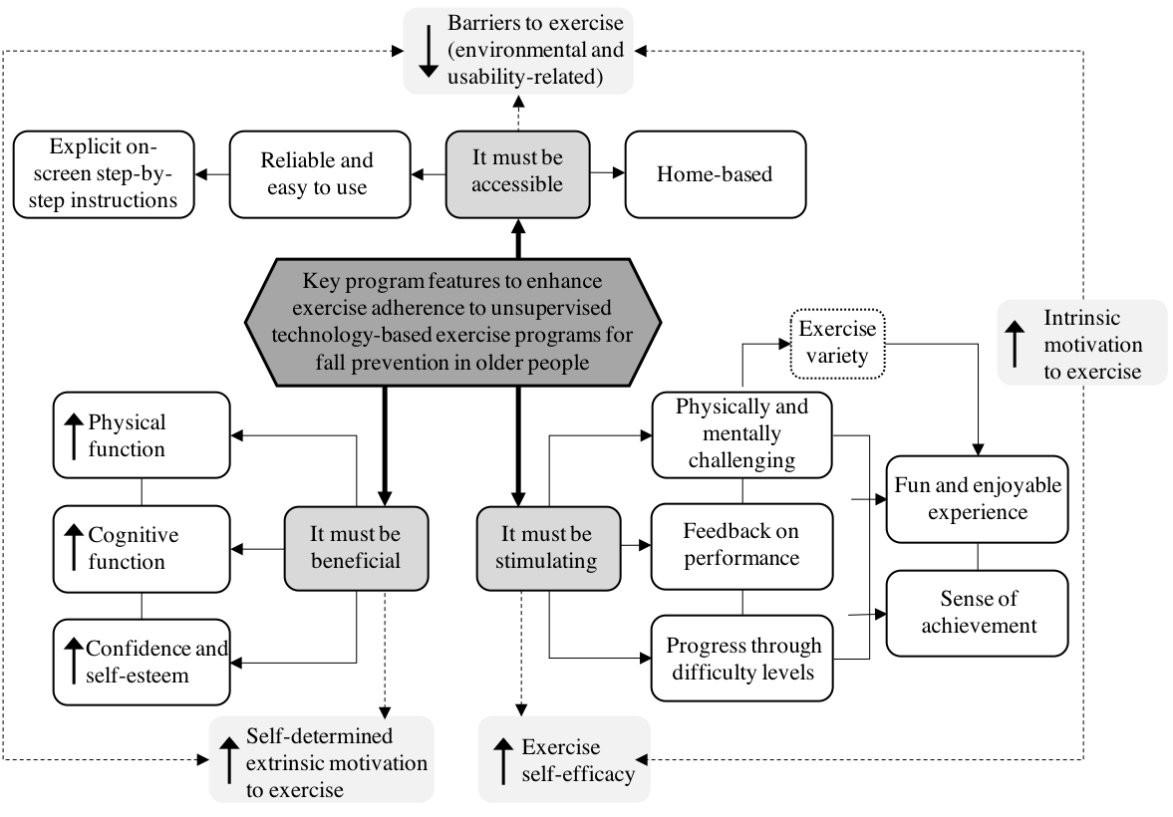
Conceptual map of the 3 main themes identified from the thematic analysis of participants’ interviews.

A conceptual map of the 3 main themes that were generated from the analysis of participants’ interviews is summarized in Figure 2. Combined, the 3 themes emphasize the importance of implementing behavioral strategies in the development of technology-based exercise programs for older adults to use at home to optimize exercise adherence. The results of this study strongly suggest that self-efficacy, attitudes and beliefs, as well as the affective response to exercise participation enhance adherence to technology-based exercise programs. Therefore, it is important to assess an individual’s needs and motivations to exercise to ensure appropriate exercise prescription.

## Discussion

### Principal Findings

This study explored older adults’ perceptions of using a technology-based exercise program unsupervised in the home setting. Findings from this study suggest that older adults are open to using technology-based exercise programs in their home. Furthermore, program uptake and adherence seem to be enhanced by (1) older adults’ perceived benefits of undertaking the program; (2) whether the program was stimulating; and (3) the perceived barriers to exercise and technology use.

Perceived benefits to health (both physical and mental health) were the most common reason for older adults to commence and continue their participation in the exercise program. Participants wanted to avoid having to rely on others and be able to care for themselves and felt that exercising would help maintain their independence and preserve their sense of self-value. This is consistent with previous literature on exercise adherence, which suggests that maintaining good health and independence and reducing health risk factors are among the most common self-determined extrinsic motivators for exercise participation in older adults [54-60]. A systematic review of perspectives on fall prevention programs found that older adults were more likely to participate in exercise interventions if they believed that these would maintain or improve their health [61]. Similarly, a study looking at perceived benefits of and barriers to adherence to home-based exercise programs also found program adherence to be influenced by the perceived effect of programs on physical and mental health [62]. Furthermore, HPM construct of perceived benefits to action also suggests that individuals are more likely to commit and engage in behaviors, such as exercise, when they anticipate personally valued benefits [53]. The findings of this study confirm this relationship between an individual’s perceived risk of future health-related problems and their commitment to exercise. Higher adherence rates were attained by participants with lower self-perceived health status, poor balance ability, fear of falling, and by participants who were hoping to obtain health-related benefits. Consistent with the findings of this study, a previous study also found poorer self-related health to predict adherence to balance exercises in older adults [63]. This motivational role of risk perception is consistent with a life-span perspective, which proposes that with advancing age, individuals become strongly driven by goals of preventive nature toward decreasing health risks and avoiding losses [64,65]. The prevalence of physical changes, health problems, and diseases can increase older adults’ perceived susceptibility to future illnesses and may lead to higher uptake of preventive health behaviors such as regular exercise participation [59]. Conversely, a lack of physical health benefits was reported by participants with higher self-perceived health status and balance ability; thus, participants reported other competing demands or preferences taking priority over following the prescribed stepping exercise program.

The importance of developing exercise programs that are stimulating to enhance exercise participation and adherence was clearly evident in this study with participants. Participants suggested a wide variety of exercises, with progressive difficulty levels, combining physical and cognitive tasks were key factors to make exercising fun, challenging, and enjoyable. Consistent with these findings, other studies have also found the enjoyment derived from exercising and the experience of personal satisfaction after exercising to be linked to older adults’ motivation to continue exercise participation [25,62,66]. Our findings are also consistent with the activity-related affect construct of HPM, which suggests that people are more likely to engage in behaviors that invoke positive feelings or emotions [53]. Self-efficacy is also a crucial psychological determinant of adoption and long-term maintenance of physical activity [67-73]. Self-efficacy refers to an individual’s judgment of their personal capabilities to execute a particular health behavior (such as engaging in exercise) and their self-confidence to perform the health behavior successfully [74]. Findings from this study suggest that *SureStep* increased participants’ self-efficacy by providing a strong sense of achievement through successful completion of the exergames and progression to higher difficulty levels. Further findings from this study suggest that presenting older adults with positive feedback in the form of praise and scores further influenced their self-efficacy to exercise and increased their motivation to try and improve their scores. These findings emphasize the importance of an immediate positive experience when performing exercise. Tailoring the exercises to the individual’s physical capacities and providing variety of exercises, opportunities for success, and positive feedback on performance are key considerations to increase program adherence.

Finally, our findings emphasize the importance of providing programs that are accessible to older adults. Participants liked the convenience of performing exercise at home, and the ability to select the amount of time spent exercising and when to exercise emerged as desirable aspects of the program. Home-based programs can reduce common barriers to exercise participation in this population, including lack of time, inability to travel, and dislike for group-based programs. By reducing perceived barriers to exercise, such programs may increase exercise participation and adherence in older adults. These findings are consistent with HPM, which postulates that perceived barriers can affect adults’ commitment to action. However, it is important to consider that not all older adults prefer to exercise at home. For some participants, the self-reliance necessary to adhere to a home-exercise program and the lack of social opportunities were a concern. A study looking at the motivational determinants of exergame participation for older adults in assisted living facilities found that social interaction encouraged older adults to use the exergame as it created competition that resulted in motivation to improve their score [75]. A previous systematic review and meta-analysis conducted on the impact of different program characteristics on adherence to home-based exercise programs found a higher proportion of older adults adhered fully to home exercise interventions that included moderate home visit support [10]. This suggests that it is equally important to promote autonomy in carrying out exercise interventions and provide support to facilitate program adherence. In this regard, technology-based exercise programs may benefit from incorporating an interactive platform through which older adults and health professionals could interact on a regular basis. This may provide opportunities to increase support while still reducing the cost associated with the provision of home-visit support. Finally, findings from this study suggest the importance of evaluating the acceptability, usability, and reliability of programs with a sample of older adults before implementing it as a home-based intervention [76]. Previous literature suggests that off-the-shelf programs that are not specifically designed for older adults may present a challenge for this population. Factors such as the pace of gameplay, the amount of graphical information, and the instructions on how to use the program can make it difficult for older adults to use these programs on their own [25,27,36,37]. *SureStep* was designed for older adults to use independently at home. The program was designed to deliver the training stimulus using a step pad connected to a computer unit and a television set. The step pad was designed to clearly indicate where the user should stand during game play, and large colored arrows were used to illustrate the different step directions the user could adopt. Two panels labeled “A” and “B” were used to access the home screen and select games and difficulty level, respectively. The program consisted of 4 games (*StepMania*, *Stepper*, *Trail-stepping,* and *Tetris*), which are modified versions of popular video games or converted neuropsychological tests. Parameters of game play including speed, color, object size, and drift rate of game elements were informed by an iterative process of design and testing involving collaboration between research staff, technologists, and older adults themselves such that the final exercise games delivered were appropriate for the physical, sensory, and cognitive abilities of an older population. As such, participants in this study did not report experiencing difficulties with factors such as the pace of gameplay or amount of graphical information displayed. However, results from our study revealed that participants with limited experience using technology experienced difficulties interacting with the program, including inability to access all available games, modify difficulty levels, and change from game to game. Older adults’ inability to use technology conveyed feelings of frustration and apathy toward the program, thus affecting exercise adherence. To facilitate a more user-friendly interface, participants suggested the use of on-screen step-by-step instructions.

Furthermore, another element that requires attention to improve future implementation of *SureStep* is the need for a greater variety of games and greater range of difficulty levels to cater for older people of varied levels of physical and cognitive function and maintain older people’s interest in the program. Results from our study showed that participants with better functional and cognitive abilities were able to successfully complete all 4 games at the highest difficulty level within the intervention period. Thus, if the aim of technologies such as *SureStep* is to provide older people with exercise programs they can successfully use at home over prolonged periods, the provision of a wider range of games with extensive difficulty levels is warranted.

### Implications Toward Design of Technology-Based Programs for Older Adults

Findings from this study provide important considerations when designing technology-based programs, which are tailored to the needs of older adults and aimed at increasing both usability and acceptability of programs, which can ultimately enhance exercise participation and long-term adherence to fall prevention interventions. Findings from our study highlight the need for designers of technologies to work closely with older adults throughout the design and development process to learn how their preferences, attitudes, and capabilities relate to technology adoption and how products and services can be designed to promote their widespread and long-term use.

On the basis of this study’s findings, future technology-based exercise programs should consider the following recommendations. First, hardware and software should be extensively tested before deployment into people’s homes to avoid system malfunction leading to feelings of frustration and apathy toward the program, which can ultimately affect program adherence. Second, user testing to evaluate the ease of use of the program interface should be carried out during the development phase of the program, and explicit step-by-step instructions should be displayed on the screen to facilitate program use, especially among those with memory problems or cognitive impairment. Third, programs should incorporate a tailored exercise progression algorithm based on the user’s performance, with increasing difficulty levels and unlocking of new games to ensure that the program remains challenging and enjoyable over time. Fourth, programs should provide a sufficient variety of games or exercises with a wide variety of difficulty levels, ideally training both cognitive and motor capacities, to maximize the perceived benefits of the program and maintain engagement. Fifth, programs that use equipment should ensure the equipment does not pose a tripping hazard, causing an increased risk of falling while stepping on or off the equipment.

### Limitations

Some limitations identified in this study need to be considered when interpreting the results. First, during the participant recruitment process, people were informed about the benefits of step training interventions in reducing fall risk factors; therefore, participants may have expected receiving health benefits from the program. Second, all participants volunteered to take part in the study knowing that if randomized to the intervention group, they would be required to use a technology-based exercise program; thus, all participants who volunteered were open to try new technologies and may have more positive attitudes toward the intervention. Third, participants were selected from the sample of older adults that returned for reassessment after a 4-month intervention period. Therefore, the interviewed participants did not include older adults who withdrew or dropped out of the study. Interviewing some of these participants may have provided further information on the perceived barriers to using a technology-based exercise program unsupervised at home. However, efforts were made to interview individuals who had stopped using the program but did not withdraw entirely from the RCT. Fourth, the structured nature of the interview questions may have prevented obtaining more in-depth information on participants’ experiences from the study. Finally, interviews were performed by the same person who provided assistance to participants during the RCT. Although this allowed for consistency and rapport building, it might have also allowed for demand characteristics, such as participants trying to provide more positive responses to the interviewer. However, data triangulation was performed using the logs from participants’ visits, telephone calls, and support required to validate the participants’ responses.

### Conclusions

Findings from this study suggest that it is feasible to use technology to deliver a home-based fall prevention intervention to community-dwelling older adults. However, to encourage uptake and long-term adherence, programs need to remain stimulating over time. Our results indicate that providing 4 different games with varying levels of difficulty did not offer sufficient variety, as over time, participants reported losing interest in the program as the novelty wore off. To maintain participants’ engagement over prolonged periods, we therefore recommend providing a greater variety of games with increasing levels of difficulty to ensure exercises remain challenging. Technologies must also be designed in ways that are accessible to older adults regardless of their previous technology exposure. This study indicates that program usability could be improved by providing on-screen step-by-step instructions as older adults with low technology familiarity as well as those with memory problems or cognitive impairment had difficulty remembering how to use the program.

In light of this evidence and in the context of optimizing adherence to technology-based exercise programs for older adults to use at home, program developers should develop exercise programs in ways that provide older adults with a fun and enjoyable experience (thus increasing intrinsic motivation to exercise), focus on improving outcomes that are significant to older adults (thus increasing self-determined extrinsic motivation), offer challenging yet attainable exercises (thus increasing perceived self-competence), provide positive feedback on performance (thus increasing self-efficacy), and are easy to use (thus reducing perceived barriers to technology use).

## Appendix

Multimedia Appendix 1 Instructional video of SureStep cognitive-motor stepping program and demonstration of study participant using SureStep.

Multimedia Appendix 2 Interview guide for qualitative interviews with older people allocated to use SureStep at completion of 4-month intervention period.

## Abbreviations

FCI: Functional Comorbidity Index
HPM: Health Promotion Model
Icon-FES: Iconographical Fall-Efficacy Scale
IPEQ: Incidental and planned exercise questionnaire for older adults
PHQ-9: Nine-item Patient Health Questionnaire
RCT: randomized controlled trial
WHODAS: World Health Organization Disability Assessment Schedule
