# Supplementary material for: An Interactive Home-Based Cognitive-Motor Step Training Program to Reduce Fall Risk in Older Adults: Qualitative Descriptive Study of Older Adults’ Experiences and Requirements
Source: JMIR Aging. 2018 Nov 30;1(2):e11975. doi: 10.2196/11975 (PMC6716489; doi:10.2196/11975)
Supplement: Multimedia Appendix 2 [file aging_v1i2e11975_app2.pdf]

General experience

1. To start with, I would like to know how your experience was while taking part in the Sure Step study.
    a. I would like you to try and remember the first few weeks you used the stepping program; can you tell me about your experience when starting to use the program?
    b. Now try and remember when you were about half way through the program (that is two months into the program); can you tell me about your experience then?
    c. Lastly, try and remember your last week using the program; can you tell me about your experience then?

    Prompts according participant's responses:
    a. How was your experience while learning how to use the program? Why do you think that was?
    b. How did you feel when using the program? What do you think made you feel like that?

Benefits

2. Can you think of any benefits you may have experienced throughout the exercise program?

    Follow-up according participant's responses:
    a. (Yes) – What benefits did you experience? How do these benefits impact your life?
    b. (No) – Why do you think that is?

Barriers and enablers to exercise adherence

3. On average, you exercised (participant's data) days per week; Depending on adherence record:
    a. I would like you to think about the different reasons that might have prevented you from exercising more.
    b. I would like you to tell me about your motivations to exercise this much.
4. Try to remember a day when you exercised using *SureStep*, can you describe what was going through your mind that made you want to exercise?
5. On the other hand, what were you thinking on the days you didn't exercise?
6. Would you see yourself continuing with an exercise program like this, let's say 3 times per week, for another year? Can you tell me more about this?

Program design

7. What would you say were the highlights of this program? Why are these important to you?
8. What would you say were the weak aspects of the program? How did this impact your experience?
9. Can you think of any suggestion to improve the program?

Closing interview

10. Is there anything else about your experience using the program, or the program itself that you feel is important and we have not yet discussed?
11. Finally, I would like to summarize what I have gathered from this interview, and I would like you to tell me if this represents what you have told me and to please clarify anything I may have misunderstood.
